# Diallylthiosulfinate (Allicin), a Volatile Antimicrobial from Garlic (*Allium sativum*), Kills Human Lung Pathogenic Bacteria, Including MDR Strains, as a Vapor

**DOI:** 10.3390/molecules22101711

**Published:** 2017-10-12

**Authors:** Jana Reiter, Natalja Levina, Mark van der Linden, Martin Gruhlke, Christian Martin, Alan J. Slusarenko

**Affiliations:** 1 Department of Plant Physiology, RWTH Aachen University, 52056 Aachen, Germany; jana.reiter@rwth-aachen.de (J.R.); martin.gruhlke@rwth-aachen.de (M.G.); 2 German National Reference Centre of Streptococci (GNRCS), University Hospital RWTH Aachen, 52074 Aachen, Germany; nlevina@ukaachen.de (N.L.); mlinden@ukaachen.de (M.v.d.L.); 3 Institute of Pharmacology and Toxicology, Medical Faculty of RWTH Aachen University, 52074 Aachen, Germany; chmartin@ukaachen.de

**Keywords:** allicin, garlic, *Allium sativum*, volatile antimicrobial agent, lung pathogenic bacteria, MDR strains, antimicrobial, *Streptococcus pneumoniae*, *Pseudomonas aeruginosa*

## Abstract

Garlic (*Allium sativum*) has potent antimicrobial activity due to allicin (diallylthiosulfinate) synthesized by enzyme catalysis in damaged garlic tissues. Allicin gives crushed garlic its characteristic odor and its volatility makes it potentially useful for combating lung infections. Allicin was synthesized (>98% pure) by oxidation of diallyl disulfide by H_2_O_2_ using formic acid as a catalyst and the growth inhibitory effect of allicin vapor and allicin in solution to clinical isolates of lung pathogenic bacteria from the genera *Pseudomonas*, *Streptococcus*, and *Staphylococcus*, including multi-drug resistant (MDR) strains, was demonstrated. Minimal inhibitory (MIC) and minimal bactericidal concentrations (MBC) were determined and compared to clinical antibiotics using standard European Committee on Antimicrobial Susceptibility Testing (EUCAST) procedures. The cytotoxicity of allicin to human lung and colon epithelial and murine fibroblast cells was tested in vitro and shown to be ameliorated by glutathione (GSH). Similarly, the sensitivity of rat precision-cut lung slices (PCLS) to allicin was decreased by raising the [GSH] to the approximate blood plasma level of 1 mM. Because allicin inhibited bacterial growth as a vapor, it could be used to combat bacterial lung infections via direct inhalation. Since there are no volatile antibiotics available to treat pulmonary infections, allicin, particularly at sublethal doses in combination with oral antibiotics, could make a valuable addition to currently available treatments.

## 1. Introduction

**Scheme 1:**
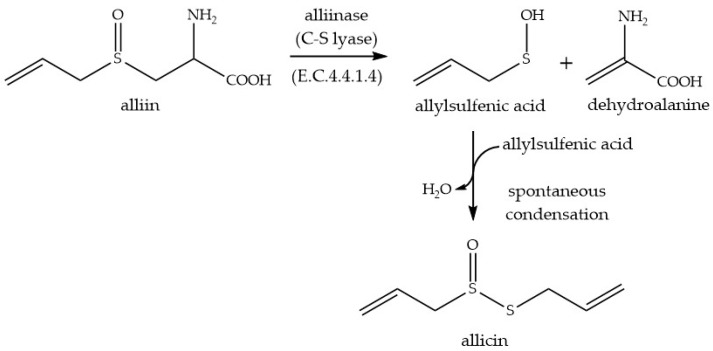
Enzyme-catalyzed biosynthesis of allicin.

The antibacterial activity associated with garlic (*Allium sativum* L.) was identified by Cavallito in 1944 as being due to diallylthiosulfinate which was given the trivial name allicin [1,2]. In vivo allicin is formed by the catalytic action of alliin-lyase (E.C.4.4.1.4) on alliin (*S*-allyl-l-cysteine sulfoxide) (Scheme 1). Enzyme and substrate are compartmentalized separately in cells and become mixed after mechanical damage. Allicin is the first major sulfur-containing volatile to be produced and gives freshly crushed garlic its typical odor. Allicin has a broad spectrum of cellular targets and it is effective against bacteria, fungi, oomycetes, and protozoa [3,4,5,6,7,8]. Allicin’s mode of action is still not fully understood [9].

**Scheme 2:**
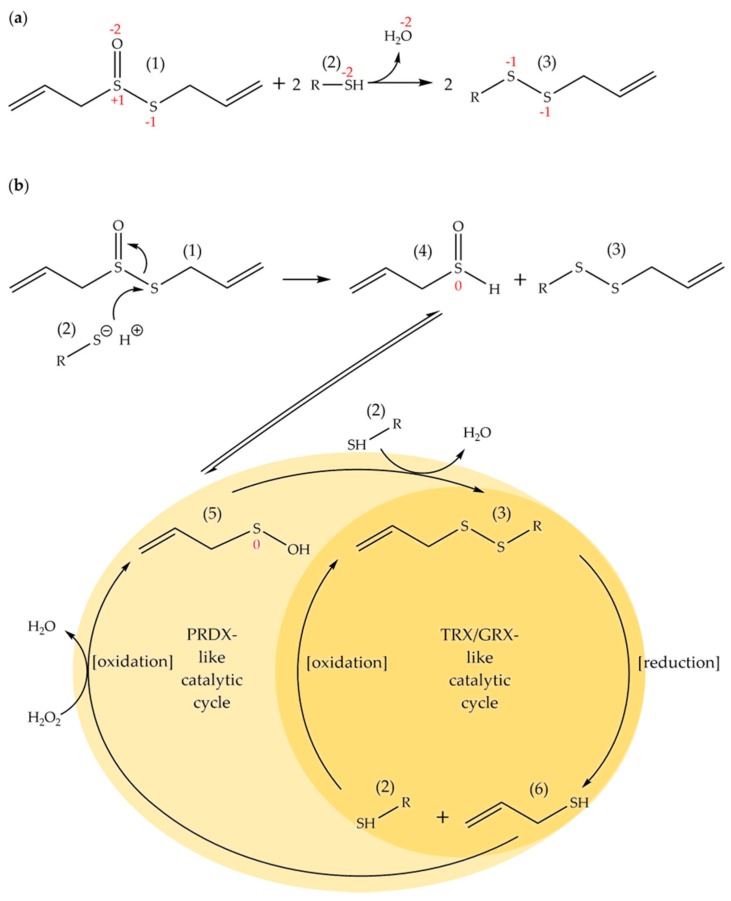
Allicin (1) is a reactive sulfur species (RSS) and undergoes a thiol-disulfide type exchange reaction in which two allicin molecules react with two thiols to produce water and two molecules of a mixed allyl disulfide (3). The reaction is shown overall in (**a**) and the formal oxidation states of the sulfur and the oxygen atoms are marked in red. In (**b**) mechanistic details are shown, breaking down the exchange reaction into component steps and showing further catalytic redox cycling of some of the components involving S-atoms with formal oxidation states of −2, −1, 0 and +1, respectively. Thus, initially, after reaction of allicin (1) with one thiol molecule (2), one allyl thial molecule (4) is formed and one molecule of a mixed allyl disulfide (3). The allyl thial is in tautomeric equilibrium with allyl sulfenic acid (5) which reacts readily with one further thiol molecule (2) to give a second mixed allyl disulfide molecule (3). These mixed allyl disulfides (3) are capable of reacting with any further thiol molecules in a standard thiol-disulfide exchange reaction shuttling interchangeably between the thiol and disulfide without producing water. If the thiols in question are cysteine residues in proteins, the protein disulfides formed (3) would be potential substrates for thioredoxins (TRX) and/or glutaredoxins (GRX) relying on reducing equivalents from NADPH-dependent thioredoxin reductases and glutathione to further cycle the reaction products. The thiols thus formed, including allyl mercaptan (6), could be catalytically re-oxidized to sulfenic acids (5) by a peroxiredoxin (PRDX) type of enzyme using H_2_O_2_, or oxidized directly but more slowly by H_2_O_2_, to yield sulfenic acids capable of further perpetuating catalytic cycling of the sulfur-containing intermediates.

However, allicin is a reactive sulfur species and undergoes a thiol-disulfide type exchange reaction (Scheme 2a) with available thiol groups, or more specifically, with thiolate ions [10,11,12]. However, in contrast to a standard thiol-disulfide exchange reaction, a molecule of water is generated from the thiosulfinate oxygen atom. The mixed disulfide formed can enter into further standard exchange reactions with fresh thiols and a redox cycling cascade can ensue, which in cells is also driven by various catalytic enzymes, e.g., thioredoxins and glutaredoxins (Scheme 2b). Thus, the effects of allicin on cellular thiol homeostasis of proteins and cellular redox buffers, such as glutathione, can be profound. For example, allicin reacts with accessible cysteines in proteins and can inactivate essential enzymes [13]. Allicin reacts with glutathione, shifts the cell redox potential to a more oxidized state and causes disulfide stress [9,14,15]. In this regard, allicin has been described as a cellular redox toxin [15]. Allicin is taken up readily by cells and has a calculated logP = 1.29 ± 0.13 [16]. The ability to pass easily through cell membranes contributes to allicin’s effectiveness as an antibiotic [17]. Furthermore, independently of its chemical reactivity, allicin’s physical properties allow it to cause transient pore formation in biological membranes and artificial lipid bilayers [18]. This effect of allicin on membranes might explain the synergistic effect of allicin with membrane-active antibiotics like amphotericin-B and polymixin-B [19,20].

Importantly, allicin is a volatile substance and can be effective against microorganisms via the gas phase. Since there are no volatile antibiotics currently available for clinical use, this makes allicin a very interesting candidate molecule. It therefore makes sense to develop new strategies using allicin-based drugs, perhaps combined with other conventional antibiotics, for direct treatment of lung infections via the pulmonary rather than the oral route. In this regard, the successful treatment of tuberculosis patients by inhalation of garlic vapor has been reported in the ‘pre-antibiotic’ era [21].

The antimicrobial effect of garlic or allicin was already documented for several human pathogenic bacteria in vitro and in animal trials [9,22,23,24]. Generally, these reports have been restricted to a few particular bacterial species, rather than a systematic treatment of a pathogenic group, for example lung pathogenic bacteria, as we report here.

**Figure 1:**
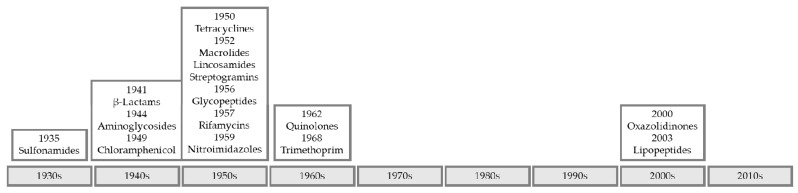
Timeline of the introduction of novel antibiotic classes into clinical practice.

Currently, the emergence of bacteria pan-resistant to current clinical antibiotics is a threat to effective treatment of infectious disease. Resistance to penicillin, the first commercially available antibiotic, was already documented in 1940 before its release into clinical practice in 1943. The appearance of resistance, rapidly following discovery or introduction into clinical practice, is also the trend for later antibiotics [25,26]. The majority of antibiotic classes and drugs were discovered before the 1970s and there have been few new discoveries reported since (Figure 1) [27,28].

Several MDR (multiple drug resistant) strains of human pathogenic bacteria e.g., *Pseudomonas aeruginosa*, *Streptococcus pneumoniae*, *Staphylococcus aureus*, *Acinetobacter baumannii*, and *Mycobacterium tuberculosis* have been reported and there is desperate need for new strategies and new classes of antibiotics [29,30,31,32,33]. In this regard, drugs like allicin, with multiple sites of action, are particularly desirable as this hinders the emergence of resistance. The seriousness of the situation is emphasized by the recent report of the death of a patient infected by a pan-resistant isolate of *Klebsiella pneumoniae* against which all 26 antibiotics allowed for clinical use in the USA were ineffective [34].

In the work reported here, we tested the antimicrobial effect of allicin as a vapor on several clinical isolates of lung pathogenic bacteria and used standardized EUCAST (European Committee on Antimicrobial Susceptibility Testing) guidelines to determine minimal inhibitory (MIC) and minimal bactericidal concentrations (MBC). The toxicity of allicin towards human lung epithelial cells and rat precision-cut lung tissue slices (PCLS) was investigated and the protective effect of glutathione was shown. The feasibility of developing allicin as part of a treatment regime for lung infections is discussed.

## 2. Results

### 2.1. Allicin Vapor Inhibits Lung-Pathogenic Bacteria

**Figure 2:**
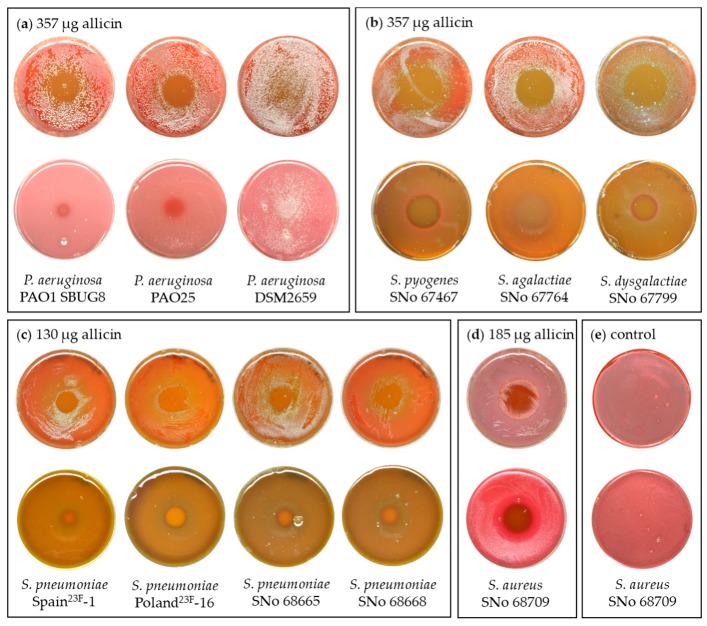
Antibacterial activity of allicin vapor. The Petri dish base with bacteria either spread on the agar surface (upper rows), or with bacteria-seeded agar (lower rows) were inverted over a 20 µL droplet of allicin solution in the center of a 9-cm diameter Petri dish lid. The absolute amount of allicin (µg) in the 20 µL droplet is stated. (**a**) The antimicrobial effect of allicin vapor on *P. aeruginosa* PAO1 SBUG8, PAO25, and the highly resistant DSM2659 strain, respectively; (**b**) The antimicrobial effect of allicin vapor on *Streptococcus pyogenes*, *S. agalactiae*, and *S. dysgalactiae equisilimlis*; respectively (**c**) The antimicrobial activity of allicin vapor on *Streptococcus pneumoniae* multi-drug resistant (MDR)-strains Spain^23F^-1 and Poland^23F^- and SNo 68665 and SNo 68668 and 16; (**d**) The antimicrobial effect of allicin vapor on *Staphylococcus aureus*; (**e**) example control plates placed over water droplets without allicin showed uniform bacterial growth.

Allicin vapor inhibited the growth of lung pathogenic bacteria over a range of concentrations. In these experiments, a 20 µL droplet of allicin solution was pipetted into the Petri dish lid and the agar-containing-base of the dish was inverted over it. Concentrations of allicin used were 110 mM, 57 mM, and 40 mM, giving total amounts of allicin in the 20 µL droplet of 357 µg, 185 µg, and 130 µg, respectively, as the source for diffusion into the air above. Petri plates with bacteria-seeded agar, or bacteria spread onto the agar surface, were incubated at 37 °C overnight. Allicin vapor diffused into the air and bacterial growth was inhibited above the droplet (Figure 2a–d). The inhibition zone was apparent as a clear circular region surrounded by a dense lawn of bacterial growth on the rest of the Petri plate. Because of technical difficulties, we were not able to determine directly the amount of allicin in the air above the droplet, but we analyzed the droplet itself and found no allicin breakdown products up to 24 h after the start of the experiment. Therefore, we conclude that allicin, and not its breakdown products, were responsible for the effects we observed. *P. aeruginosa* strains PAO1 and PAO25 showed inhibition but *P. aeruginosa* DSM2659, which was very resistant to allicin in the EUCAST MIC and MBC tests, showed no inhibition (Figure 2a, Table 1). The Streptococcus pyogenes, *S. agalactiae* and *S. dysgalactiae* equisilimlis strains tested all showed inhibition zones above the allicin droplets (Figure 2b) and swabs taken from inhibition zones and streaked onto fresh medium showed no bacterial growth, suggesting a bactericidal rather than a bacteriostatic effect. Allicin vapor was effective against clinical isolates of *Streptococcus pneumoniae*, including MDR-strains Spain^23F^-1 and Poland^23F^-16 (Figure 2c). Furthermore, allicin vapor was effective against a *Staphylococcus aureus* isolate (Figure 2d). Uniform bacterial growth was observed over the whole plate in controls placed over water droplets without allicin, as can be seen in Figure 2e for *S. aureaus*.

### 2.2. MIC and MBC

The growth of the majority of *Pseudomonas*, *Streptococcus*, and *Staphylococcus* isolates was completely inhibited by 64 µg/mL allicin (Table 1). *S. pyogenes* SNo 67467, *S. pneumoniae* SNo 68668, and *S. aureus* ATCC 43300 were completely inhibited by 32 µg/mL allicin and all *A. baumannii* isolates were completely inhibited by 16 µg/mL. *K. pneumoniae* isolates were slightly more resistant, with a MIC of 128 µg/mL. *P. aeruginosa* DSM2659 showed high resistance to allicin (MIC = 512 µg/mL) compared to *P. aeruginosa* PAO1 SBUG8 and PAO25 (MIC = 64 µg/mL). MDR and non-MDR *S. pneumoniae* strains tested were equally susceptible to allicin and showed MICs from 32 to 64 µg/mL allicin and MBCs from 64 to 128 µg/mL allicin, respectively (Table 1).

In comparison to conventional antibiotics, the MICs and MBCs for allicin were generally higher, both in terms of µg/mL and absolute concentrations in µM (Table 2). Thus, except for the MDR strains, the clinical isolates of *S. pneumoniae* were susceptible to all tested antibiotics at <1 µg/mL. The MDR *S. pneumoniae* isolates were resistant to erythromycin and clindamycin (MICs > 256 µg/mL) and for these MDR strains, allicin, including in absolute µM terms, compared favorably with those antibiotics.

### 2.3. Cytotoxicity of Allicin to Mammalian Cells

**Figure 3:**
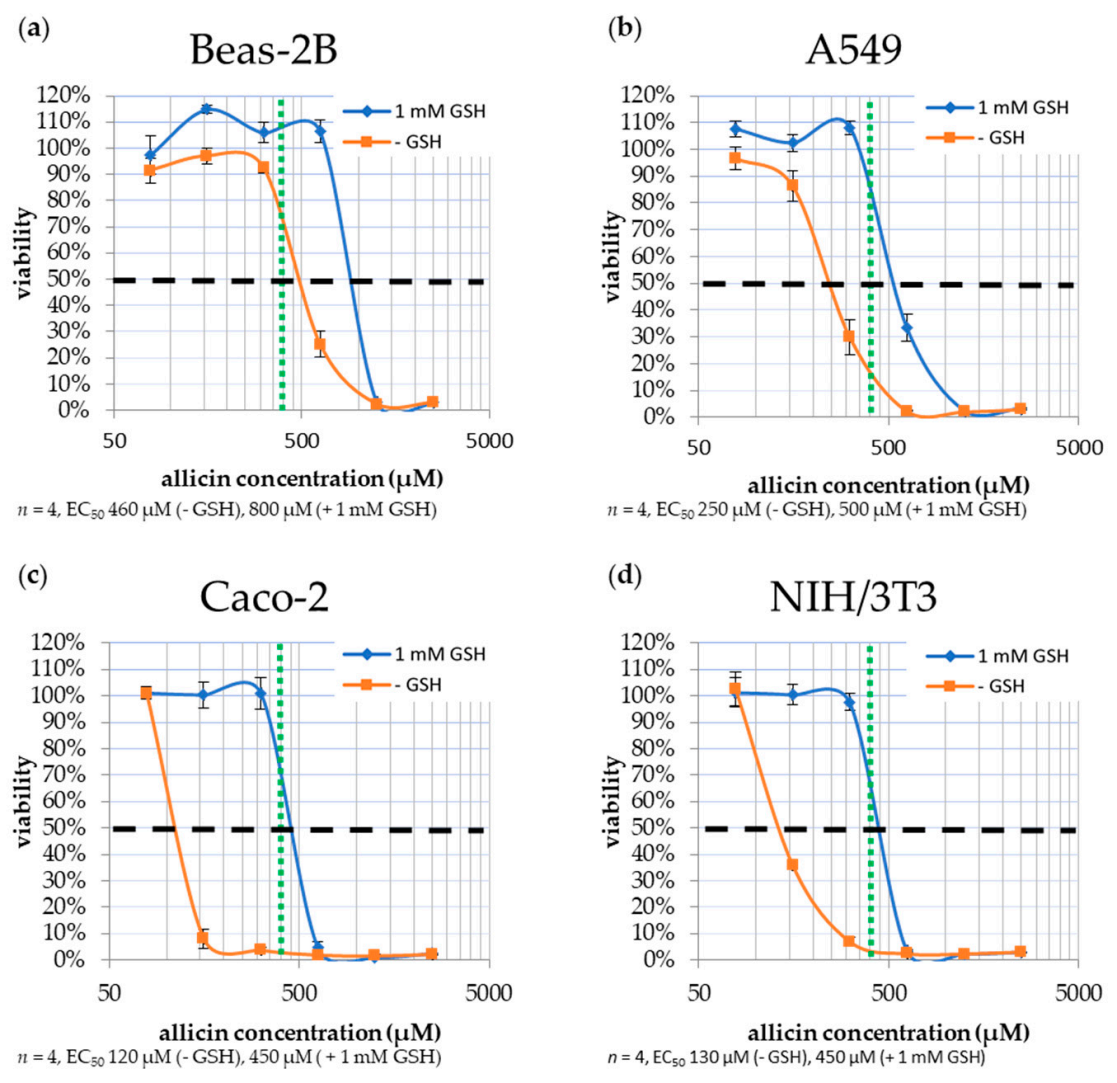
Susceptibility of mammalian cell lines to allicin. Cell cultures were stressed for one hour by exposure to allicin at different concentrations, either in the absence or presence of 1 mM reduced glutathione (GSH). Cell viability was determined with the MTT-(3-(4,5-dimethylthiazol-2-yl)-2,5-diphenyltetrazolium bromide)-test. The half maximal effective concentration (EC_50_) for allicin was determined and is marked with a horizontal dashed line (- - - - -). The MIC value for the majority of tested bacteria (390 µM allicin, 64 µg/mL) is marked with a vertical dotted line (………). (**a**) Beas-2B (SV40-immortalized human bronchial epithelial cells); (**b**) A549 (human epithelial lung carcinoma); (**c**) Caco-2 (human epithelial colon tumor) and (**d**) NIH/3T3 (murine embryonic fibroblast), respectively.

The effect of allicin on mammalian cell cultures was tested with two human lung epithelial cell lines (A549 and Beas-2B), a human colon cancer epithelial cell line (Caco-2), and murine embryonic fibroblasts NIH/3T3. Two-week-old cell cultures in 96-well plates were incubated with allicin for one hour. After challenge, remaining allicin was titrated out by reaction with cysteine and the plates were incubated for 23 h. The experiment was repeated with 1 mM GSH in the medium since this partially simulates the situation for cells in the body, which are continually surrounded by body fluids containing GSH. Cell viability was determined with the MTT (3-(4,5-dimethylthiazol-2-yl)-2,5-diphenyltetrazolium bromide) test (Figure 3).

The human lung epithelia cell lines Beas-2B and A549 showed EC_50_ values of 460 and 250 µM allicin (75 and 41 µg/mL, respectively) which increased to 800 and 500 µM (130 and 81 µg/mL, respectively) in the presence of 1 mM GSH (Figure 3a,b). Human Caco-2 cells and NIH/3T3 murine fibroblasts were particularly susceptible to allicin, showing an EC_50_ of 120 and 130 µM (19.5 and 21 µg/mL, respectively) which increased to 450 µM (73 µg/mL) in both cases in the presence of 1 mM GSH (Figure 3c,d).

### 2.4. Cytotoxicity of Allicin to Rat PCLS

PCLS were incubated with allicin solutions for one hour with or without 1 mM GSH. Cell viability was determined with LDH (lactate dehydrogenase) and WST (water soluble tetrazolium) tests. In the absence of GSH cell in the lung slices were extremely sensitive to allicin with EC_50_ values of 14.7 µM in the LDH-test and 14.4 µM in the WST-test, (~2.5 µg/mL, respectively). The EC_50_ value increased in the presence of 1 mM GSH to 656 µM (106 µg/mL) in the LDH-test and 280 µM (45 µg/mL) in the WST-test, respectively (Table 3).

## 3. Discussion

We have previously shown in agar diffusion assays that allicin compared well on a mol-for-mol basis with ampicillin and kanamycin against *E. coli* [35], and there have been numerous individual reports that allicin, often in garlic juice rather than the pure substance, was effective against human pathogens, including MDR strains and MRSE [9,22,36]. However, because allicin reacts with thiols, it is rapidly titrated out by glutathione in bodily fluids and it is therefore not suitable for clinical application via the oral route [37]. Subcutaneous application of garlic extract has been used in mice to treat lung infections with *Pseudomonas aeruginosa* and led to the disappearance of the bacteria in the infected lungs [23]. However, scaling up for application in humans would require approximately 50 garlic bulbs per day per person and the authors concluded that this was not feasible. In clinical trials on the effect of consuming garlic capsules on cystic fibrosis patients, although positive tendencies were seen, treatment didn’t produce significant improvements [37]. Because of the problems of attaining effective concentrations of allicin where it is needed when it is consumed orally, direct inhalation in the case of lung infections seems an attractive alternative. The successful treatment of tuberculosis patients by inhalation of garlic vapor has been reported [21].

Allicin vapor was inhibitory to the growth of the clinical isolates tested with the exception of the very resistant *P. aeruginosa* DSM2659 strain (Figure 2). These results confirm ‘proof of principle’ that it could be possible to administer allicin via the pulmonary route rather than the oral route to combat lung infections. Furthermore, it may be possible to use sublethal concentrations of allicin in conjunction with other antibiotics. A synergistic action of allicin with beta-lactams (cefazolin, oxacillin, and cefoperazone) was shown to decrease the MIC to *P. aeruginosa* and *Staphylococcus* spp. [24]. A synergistic action between allicin and the antifungals amphotericin-B and polymixin-B has also been reported [19,20].

MIC for the bacteria tested ranged from 32 to 128 µg/mL allicin with the majority of strains being completely inhibited by 64 µg/mL allicin, except for *P. aeruginosa* DSM2659 which had an MIC of 512 µg/mL allicin. A similarly high MIC was found for *P. aeruginosa* DSM50071 which might suggest that allicin is likely to be less effective against *P. aeruginosa* infections than against other pathogens [9]. Encouragingly, the MDR strains of *S. pneumoniae* were equally as susceptible to allicin as the non-MDR strains (Table 1). The majority of clinical isolates tested had MBCs from 64 to 128 µg/mL allicin, whereas *P. aeruginosa* DSM2659 again showed greater resistance to allicin than other pathogenic strains (MBC = 1024 µg/mL).

Despite the MDR strains being as susceptible to allicin as the non-MDR strains, the overall performance of allicin compared to clinical antibiotics was poor (Table 2). Nevertheless, for those antibiotics against which the MDR strains were resistant, the quantitative comparison with allicin was in some cases favorable, for example with *S. pneumoniae* CSR^14^-10, S. Africa^19A^-13 and Poland^23F^-16 and erythromycin and clindamycin MIC > 256 µg/mL and for allicin 64 µg/mL. On this basis, allicin might be considered as a treatment for MDR strains. Where there is allergy to a particular antibiotic, allicin might also be considered.

Mammalian cells proved highly susceptible to allicin with EC_50_ values for the lung epithelial cell lines near the 64 µg/mL value taken as the general MIC for most bacteria (Figure 3 and Table 1). Caco-2 and NIH/3T3 cells were even more susceptible than lung epithelial cells with EC_50_ of 19.5 and 21 µg/mL, respectively, well below the MIC of 64 µg/mL allicin for bacteria. Incorporating 1 mM GSH into the growth media raised the apparent EC_50_ value in all cases, however, this is because the effective dose of allicin is reduced in a simple reaction with the GSH to make *S*-allylmercaptoglutathione [14].

Rat PCLS were even more susceptible to allicin than cells in culture with EC_50_ values of ~14.5 µg/mL allicin in the LDH and WST tests. The EC_50_ values were raised more than seven-fold to 106.4 µg/mL allicin (LDH test) and three-fold 45.5 µg/mL allicin (WST test) when the rat slices were supplied with 1 mM GSH (Table 3). Hess et al. [38] recently showed that the toxicity of a range of industrial chemicals in PCLS correlated well with in vivo aerosol application in rats for compounds stable in water. Therefore, the results we report here are not encouraging for developing allicin for treating lung infections, however, these in vitro data must be considered in the context of the in vivo situation. By virtue of their function, lung epithelial cells have to be protected against oxidative insults and the cells are well and continually supplied with GSH (1 mM GSH in whole blood) and have high levels of GSH and other intrinsic antioxidant systems [39,40,41,42]. We have shown that GSH levels in *E. coli* drop significantly during allicin stress and we have shown that GSH levels are important for the resistance of yeast to allicin with mutants deficient in GSH synthesis and metabolism being particularly susceptible [9,43]. This intrinsic protection against allicin due to endogenous GSH and other low molecular weight thiols in the bacteria themselves, must be overcome before cells will be inhibited by allicin. The GSH concentration in alveolar fluid (epithelial lining fluid, ELF) has been reported to be 0.4 mM, 140 times more than in plasma [44] and in rat lungs 2 mM GSH has been reported [42]. This is completely different to the in vitro situation for cells in culture which are cut off from continual blood circulation. Mostly, lung-pathogenic bacteria colonize the gel layer of the mucus, which is separated from the epithelial cells by an additional sol layer [45]. Therefore, it is very likely that bacteria colonizing the bronchial and alveolar air spaces have a less GSH-rich environment than the epithelial cells lining the lungs. High levels of GSH were found in ELF, which mainly consists of mucus and cells. As the pathogens (bacteria) would be on the surface of the mucus they would be more exposed to the inhaled allicin, whereas the epithelial cells would be more protected. This increased exposure of lung cells, but not the invading bacteria, to GSH in the bathing fluids may help with differential susceptibility of bacteria vs. lung cells in clinical treatment of infection. This question can only be clarified by experiments in animal models, which at present are beyond the scope of this investigation. Nevertheless, there are historical precedents for the successful treatment of lung infections by garlic preparations. Thus, Minchin [21] used a specially designed mask to treat tuberculosis patients twice daily by one-hour inhalation periods with crushed garlic preparations containing ‘oleum allii’. Minchin also reported using oleum allii inhalation prophylactically “in the homes of, and by the members of, families notably affected by pulmonary tuberculosis”. These pioneering studies dropped out of focus as streptomycin, without the unpleasant smell of allicin, was introduced in 1944 and used as a treatment for tuberculosis.

## 4. Materials and Methods

### 4.1. Allicin Synthese

Allicin was synthesized by oxidation of diallyl disulfide (DADS) with H_2_O_2_ as reported previously [46].

### 4.2. Bacteria

The *Pseudomonas aeruginosa* strains PAO1 SBUG8, PAO25, and DMS2659 were from the culture collection of the Institute for Applied Microbiology (IAM, RWTH Aachen). The *Streptococcus pneumoniae strains* (MDR strains: Spain^23F^-1 (PMEN-1), CSR^14^-10 (PMEN-10), S. Africa^19A^-13 (PMEN-13), Poland^23F^-16 (PMEN-16) (http://www.pneumogen.net/pmen), clinical isolates: SNo 67715, SNo 68668, SNo 68665), *Streptococcus pyogenes* (clinical isolate SNo 67467), *Streptococcus dysgalactiae equisilimlis* (clinical isolate SNo 67799, SNo 73742), *Streptococcus agalactiae* (clinical isolate SNo 67764, SNo 69235), *Staphylococcus aureus* (ATCC 43300 and clinical isolate SNo 68709), *Klebsiella pneumoniae strains* (clinical isolates SNo 45412, SNo 45413), and *Acinetobacter baumannii* (clinical isolates SNo 45541, SNo 45757, SNo 45760) were from the culture collection of the German National Reference Center for Streptococci (GNRCS), University Hospital, RWTH Aachen.

### 4.3. Antibiotic Activity of Allicin Vapor

Bacteria were grown over night at 37 °C on blood-CASO-agar plates. Bacterial colonies were resuspended in 5 mL MHB2 medium to an optical density at 600 nm (OD_600_) = 0.2. Defibrinated sheep blood was added to 25 mL molten CASO-agar at 50 °C (5% *v*/*v*), mixed with 300 µL of bacterial suspension and poured immediately into a Petri dish to make bacteria-seeded agar plates. Bacteria were either spread onto the agar surface. Different concentrated allicin solutions (20 µL) were pipetted onto the Petri dish lid and the solidified agar plate with bacteria was placed inverted over the lid. The amount of allicin in the droplet is indicated in Figure 2. Bacterial growth was scored after incubation over night at 37 °C.

### 4.4. MIC and MBC Determination

Susceptibility testing was performed following the EUCAST guidelines together with the GNRCS using the broth dilution method in 96-well microtiter plate format [47].

Bacteria were grown over night at 37 °C on blood-CASO-agar plates (CASO-Agar, Carl Roth, 5% defibrinated sheep blood Thermo Fischer Scientific GmbH, oxoid limited, Basingstoke, UK), *P. aeruginosa* without blood. Bacterial colonies were resuspended in 5 mL cation-adjusted Müller–Hinton Broth 2 (MHB2, Sigma-Aldrich, St. Louis, MO, USA) up to 1 McFarland. Double concentrated MHB2 (2.2 mL), 141 µL lysed horse blood (Thermo Fischer Scientific GmbH, oxoid limited, Basingstoke, UK) and 10 µL bacterial suspension were mixed and kept on ice until pipetting into microtitre plates.

A two-power dilution series of allicin in water (2–2048 µg/mL = 12 µM–12.6 mM) was prepared and 50 µL was mixed with 50 µL of bacteria suspended in blood-MHB2 medium (see above) in 96-well plates so that the final bacterial suspension in the test wells was 0.5 McFarland. Plates were covered with air-permeable, self-adhesive cling film (Carl Roth GmbH, Karlsruhe, Germany) and incubated at 37 °C for 20 h without shaking. The lowest allicin concentration without growth gave the MIC. For the determination of MBC 10 µL from each well were pipetted onto agar plates (see above) and incubated at 37 °C overnight. The lowest concentration without bacterial growth was the MBC.

### 4.5. Effect of allicin on Mammalian Cells

Lung epithelial cell lines Beas-2B (SV40-immortalized human bronchial epithelial cells) and A549 (human epithelial lung carcinoma), Caco-2 (human epithelial colon tumor) and fibroblast NIH/3T3 (murine embryonic fibroblast) cells were tested for their sensitivity to allicin. Beas-2B, A549 and Caco-2 were cultivated in DMEM medium with penicillin/streptomycin 1% (*v*/*v*) (each 10,000 U/mL, Lonza, Verviers, Belgium) and fetal bovine serum (FBS) 10% (*v*/*v*) (Sigma-Aldrich, St. Louis, USA). NIH/3T3 cells were cultivated in Roti®-CELL RPMI-1640 (Carl Roth GmbH, Karlsruhe, Germany) media with penicillin/streptomycin 1% (*v*/*v*) and FBS 10% (*v*/*v*). Cells were cultivated in TC (tissue culture) dishes (60 mm, Sarstedt, Nümbrecht, Germany) at 37 °C and 5% atmospheric CO_2_ and sub-cultured weekly. For experiments, the cell culture was grown directly in 96-well plates (100 µL per well).

Cells were exposed to allicin (diluted in medium) for 1 h (controls with medium only). Allicin concentrations between 78 and 2500 µM (12.7–406 µg/mL) (two-power dilution series) were tested. In a further experiment, cells were incubated with GSH 1 mM (Sigma-Aldrich, St. Louis, MO, USA). After incubation, unreacted allicin was titrated out by the addition of 100 µL cysteine solution 5 mM (AppliChem GmbH, Darmstadt, Germany, dissolved in media). Cells were post-incubated for 23 h at 37 °C and 5% CO_2_. Cell viability was tested with MTT (3-(4,5-dimethylthiazol-2-yl)-2,5-diphenyltetrazolium bromide, Carl Roth GmbH, Karlsruhe, Germany). 50 µL of MTT 0.5% (*w*/*v*) dissolved in phosphate-buffered saline (PBS) was added to each well and the plate incubated for 3 h at 37 °C and 5% CO_2_. Cells were lysed by adding 100 µL isopropanol and the A570 subtracted from A630 automatically in the plate reader (TriStar2 LB942, Berthold Technologies, Bad Wildbad, Germany). The results are presented as half maximal effective concentration (EC_50_) showing a 50% reduction in MTT response.

### 4.6. Effect of Allicin on Rat PCLS

Female Wistar rats (*Rattus norvegicus*) (270 ± 10 g) were purchased from Charles River (Sulzfeld, Germany). Animal studies were approved by the Landesamt für Natur, Umwelt und Verbraucherschutz Nordrhein-Westfalen (ID: 8.87-51.05.20.10.245, 16 April 2013) and performed following the Directive 2010/63/EU of the European Parliament. Animals were euthanized with a lethal dose of pentobarbital (60 mg/kg). After confirming that the animals did not show any reflexes, the abdomen was opened and the lung was removed. The lung was filled via the trachea with 37 °C 1.5% (*w*/*v*) low-melting agarose and the lung was transferred to ice-cold PBS. After the agarose solidified, the lung was sliced first into 10 mm thick cylinders and afterwards into 250 µm thin slices with a Krumdieck tissue slicer (Alabama Research and Development, Munford, TN, USA). The slices were placed in a 24-well plate (two slices per well). One well per treatment and per animal was used. Each experiment was performed with three animals.

PCLS were incubated at 37 °C and 100% humidity in MEM (minimal essential medium) supplemented with CaCl_2_ 1.8 mM, MgSO_4_ 0.8 mM, KCl 5.4 mM, NaCl 116.4 mM, glucose 16.7 mM, NaHCO_3_ 26.1 mM, Hepes 25.17 mM, sodium pyruvate 1 mM, glutamine 2mM, MEM amino acids and vitamin mix. Agarose was removed from the slices by replacing the medium every 30 min for two hours and then every 60 min for the next 2 h before being used for experiments [48].

The slices were incubated for 1 h with allicin (0.1 µM, 1 µM, 10 µM, 33 µM, 100 µM, 333 µM, and 1 mM) (16.2 ng/mL, 162.3 ng/mL, 1.6 µg/mL, 5.3 µg/mL, 16.3 µg/mL, 54.0 µg/mL, 162.3 µg/mL). Allicin was removed after incubation by washing the slices 3 times with PBS and post-incubating the tissue slices for 23 h.

Cell viability was determined using the lactate dehydrogenase activity test (LDH cytotoxicity detection kit, Roche) and via measurement of the mitochondrial metabolic activity with WST-1 (water soluble tetrazolium) kit (Roche Diagnostics GmbH, Mannheim, Germany).

## 5. Conclusions

In conclusion, allicin in the gas phase is antimicrobial towards the majority of pathogenic isolates tested, including antibiotic resistant strains. However, the relatively low differential sensitivity to allicin in vitro between animal and bacterial cells in liquid culture suggests that allicin alone may not be a suitable alternative to conventional antibiotics to treat lung infections, although the ameliorating effect of continual GSH supply to lung cells in vivo is an unknown variable. Nevertheless, in light of the historical precedents and the increasingly urgent need for new alternatives [49], the use of allicin at sublethal doses in combination with other antibiotics merits further investigation.

## Figures and Tables

**Table 1:** Minimal inhibitory concentration (MIC) and minimal bactericidal concentration (MBC) in µg/mL for allicin. The MICs and MBCs were determined following EUCAST (European Committee on Antimicrobial Susceptibility Testing) guidelines. MDR strains are shown in bold.

Organism	MIC (µg/mL)	MBC (µg/mL)
***S. pneumoniae* Spain^23F^-1**	64	128
***S. pneumoniae* CSR^14^-10**	64	128
***S. pneumoniae* S.Africa^19A^-13**	64	128
***S. pneumoniae* Poland^23F^-16**	64	64
*S. pneumoniae* SNo 67715	64	64
*S. pneumoniae* SNo 68665	64	64
*S. pneumoniae* SNo 68668	32	64
*K. pneumonia* SNo 45412	128	128
*K. pneumonia* SNo 45413	128	128
*A. baumannii* SNo 45541	16	32
*A. baumannii* SNo 45757	16	32
*A. baumannii* SNo 45760	16	32
*P. aeruginosa* PAO1 SBUG8	64	128
*P. aeruginosa* PAO25	64	256
*P. aeruginosa* DSM2659	512	1024
*S. aureus* SNo 68709	64	256
*S. aureus* ATCC 43300	32	512
***S. pyogenes* SNo 67467**	32	64
*S. dysgalactiae* SNo 67799	64	1024
*S. dysgalactiae* SNo 73742	64	1024
*S. agalactiae* SNo 67764	64	128
*S. agalactiae* SNo 69235	64	64

**Table 2:** Comparison of MICs for allicin and conventional antibiotics. MDR strains are shown in bold. The horizontal coloured bar, ranging from dark green through yellow to orange and red, indicates increasing concentration of test substances.

	MIC (µg/mL)	Allicin	PEN	FOT	ERY	CLIN	TET	CHL	SXT	AMOX	LEVO	MXF	VAN
Organism													
***S. pneumoniae*** **Spain^23F^-1**		64	1	1.5	0.025	0.19	64	16	2	NT	NT	NT	NT
***S. pneumoniae*** **CSR^14^-10**		64	8	1	≥256	≥256	48	32	0.25	NT	NT	NT	NT
***S. pneumoniae*** **S.Africa^19A^-13**		64	1.5	0.5	≥256	≥256	48	24	4	NT	NT	NT	NT
***S. pneumoniae*** **Poland^23F^-16**		64	8	4	≥256	≥256	64	16	1.5	NT	NT	NT	NT
*S. pneumoniae* SNo 67715		64	4	2	≤0.12	≤0.12	16	≤2	1	4	1	≤0.25	≤0.5
*S. pneumoniae* SNo 68668		32	≤0.015	≤0.015	≤0.12	≤0.12	0.25	≤2	≤0.25	≤0.015	1	≤0.25	≤0.5
*S. pneumoniae* SNo 68665		64	≤0.015	≤0.015	≤0.12	≤0.12	0.25	≤2	≤0.25	≤0.015	1	≤0.25	≤0.5
***S. pyogenes*** **SNo 67467**		32	≤0.015	≤0.015	≥256	64	64	≤2	≤0.25	≤0.015	1	≤0.25	≤0.5
*S. dysgalactiae* SNo 67799		64	≤0.015	≤0.015	≤0.12	≤0.12	16	≤2	≤0.25	0.03	1	≤0.25	≤0.5
*S. agalactiae* SNo 67764		64	0.03	0.06	≤0.12	≤0.12	64	≤2	≤0.25	0.06	1	≤0.25	≤0.5
drug concentration (mg/L)													
0.06		0.12	0.25	0.5	1	2	4	8	16	32	64	128	256

NT, not tested; PEN, penicillin; FOT, cefotaxime; ERY, erythromycin; CLIN, clindamycin; TET, tetracyclin; CHL, chloramphenicol; AMOX, amoxicillin; LEVO, levofloxacin; MXF, moxifloxacin; VAN, vancomycin.

**Table 3:** Sensitivity of rat lung tissue slices to allicin. EC_50_ values for allicin in the lactate dehydrogenase (LDH) and water soluble tetrazolium (WST) tests either in the absence or presence of 1 mM GSH.

	EC_50_ Allicin	
	without GSH	with 1 mM GSH
LDH-test	14.7 µM (2.4 µg/mL)	655.9 µM (106.4 µg/mL)
WST-test	14.4 µM (2.3 µg/mL)	280.5 µM (45.5 µg/mL)
